# An assessment of Parkinson’s disease medication treatment patterns in the Medicaid population

**DOI:** 10.1016/j.prdoa.2021.100109

**Published:** 2021-09-27

**Authors:** Michael Johnsrud, Kristin Richards, Steve Arcona, Rahul Sasané, Matthew Leoni

**Affiliations:** a TxCORE (Texas Center for Health Outcomes Research and Education), The University of Texas at Austin, 2409 University Avenue, Austin, TX 78712, USA; b Cerevel Therapeutics, 222 Jacobs Street, Suite 200, Cambridge, MA 02141, USA

**Keywords:** Parkinson’s disease, Medicaid, Medication adherence, Levodopa, Dopamine agonist

## Abstract

**Introduction:**

Most Parkinson’s disease (PD) medication adherence studies have focused on patients with commercial or Medicare health insurance coverage. However, less is known regarding medication treatment patterns within the Medicaid population.

**Methods:**

This retrospective cohort study utilized 2011–2019 administrative healthcare claims from 7 state Medicaid programs. We compared newly diagnosed patients with PD started on either levodopa or a dopamine agonist (DA). Baseline comorbidities were compared. Outcomes were assessed during a 12-month post-index observation period, and included total medication days, proportion of days covered (PDC), adherence status, persistence to initiating PD medication, and time to non-persistence of initiating PD medication.

**Results:**

Our study sample of 805 Medicaid patients had an average age of 54.1 years, with 52.0% being female. Levodopa was the predominant PD medication at initiation (75.4%). Roughly half of patients had a baseline depressive disorder and nearly 40% had an anxiety disorder. Levodopa patients had a significantly higher PDC compared to DA patients (0.621 vs. 0.546, p = 0.007). An adjusted logistic regression model showed no significant difference in the number of adherent patients between the two groups (p = 0.058). An adjusted Cox proportional hazards model controlling for demographic and baseline variables showed a 26% lower risk of non-persistence for levodopa patients versus DA patients (HR 0.740, CI 0.597–0.917, p = 0.006).

**Conclusions:**

Adherence and persistence rates were suboptimal following initiation of either levodopa or DA medication for patients with PD in Medicaid programs, though rates were better for those initiated on levodopa.

## Highlights

• There is a lack of published research assessing Parkinson’s disease medication treatment patterns in Medicaid programs.
• Roughly half of Parkinson’s disease patients in our study who initiated on levodopa or a dopamine agonist had a baseline depressive disorder.
• Adherence rates are suboptimal following initiation of either levodopa or a dopamine agonist for patients with PD in Medicaid programs.
• Adherence and persistence rates were better for patients initiating on levodopa.

## 1 Introduction

Parkinson’s disease (PD) represents a neurologic disorder that has been growing in prevalence and in its impact on patient disability. The global burden of the disease has doubled over the last 20 years due to factors such as population aging and increasing life expectancy [1]. Recent estimates of PD prevalence in the US show a growth between 2010 and 2020 from 680,000 to 930,000 individuals aged 45 or older, with projections of 1.2 million by 2030 [2]. The fiscal burden of PD in the US, including both direct and indirect costs, was estimated as high as $23 billion in 2002 [3]. Given the progressive nature of PD, the effects of the disease’s motor complications are often accompanied by other disorders such as depression, anxiety, and dementia, further impacting the quality of life of patients and their caregivers [4].

The most common initial treatment modality for PD for more than 20 years has been levodopa or a dopamine agonist (DA) taken daily [5], [6]. Treatment initiation strategy for PD has been a subject of debate for years with some clinicians preferring to delay the longer-term side effects of levodopa by initiating patients on a DA. However, there is evidence that this trend is shifting back to initiating patients with levodopa [7].

Adherence to and persistence with prescribed medications are important treatment goals for PD patients to optimize efficacy in maintaining adequate motor functioning and quality of life [8]. However, research results indicate that medication adherence to the most common PD treatments is suboptimal. With adherence being defined as having adequate medication supply at least 80% of the time over the course of a year, studies have reported that only 33% to 54% of patients are adherent to their PD therapy [9], [10], [11], [12], [13].

## 2 Objective

Most PD medication adherence studies have focused on patients whose primary insurance coverage is represented by commercial [9], [10], [13] or Medicare [12] health plans. However, less is known regarding treatment patterns for PD patients within the Medicaid population. State Medicaid programs are important for payers for Parkinson’s-related healthcare as many are diagnosed before the age of 65 and qualify for Medicaid due to income losses (e.g., early retirement, increasing healthcare costs) [14]. Tarrants et al. studied PD medication adherence using IMS health prescription claims data for patients with Medicaid and commercial insurance, but the proportion of and outcomes specifically for Medicaid patients were not reported [11]. No other PD medication adherence studies in a Medicaid population were identified. Therefore, our research objective was to characterize treatment patterns for patients initiating medication therapy for PD enrolled in state Medicaid programs, with a specific focus on comparisons of medication adherence and persistence between patients newly started on levodopa versus DAs.

## 3 Methodology

### 3.1 Study design and data source

This retrospective cohort study utilized secondary data comprised of de-identified administrative healthcare claims from medical, pharmacy, and enrollment files from 7 state Medicaid programs. The data included Medicaid claims from both traditional fee-for-service and managed care delivery designs.

Data from September 2011 through June 2019 were used for the study. Within our study sample, we assessed claims data from a single state with available service dates between September 2011 and August 2016. We assessed the remaining states’ claims data with available service dates between January 2012 and June 2019. A pooled analysis file was created for the 7 states.

The index date was defined as the first prescription for levodopa or a DA (bromocriptine, cabergoline, ropinirole, pramipexole, or rotigotine) allowing for a 12-month pre-index period to determine baseline variables, as well as a 12-month post-index period following the index date to measure medication treatment patterns. Patients were assigned to either a levodopa or DA cohort based on the type of PD medication represented by the index prescription claim. The Institutional Review Board (IRB) at The University of Texas at Austin provided approval for this study.

### 3.2 Study sample

Patients were included if they: (1) were continuously enrolled in Medicaid (with medical and pharmacy coverage) for at least 12 months prior to and following the index date; (2) had evidence of a PD diagnosis (International Classification of Disease, Ninth Revision (ICD-9) of 332.0/332.1 or ICD-10 of G20/G21) on at least two separate occasions during the baseline or observation period; (3) had no evidence of a prescription claim for levodopa or a DA in the 12-month baseline period; and (4) were at least 18 and less than 64 years of age at the index date. Patients with evidence of dual-eligible Medicare/Medicaid status were excluded to ensure data capture for prescription utilization within the Medicaid program.

### 3.3 Variables and outcomes

The primary variable of interest was the index medication (levodopa or DA) which dictated the study cohort assignment. Patient age and gender were determined at index. Baseline comorbid conditions of interest (depressive disorder, anxiety disorder, cognitive impairment, dementia, psychosis, and insomnia) were identified by assessing inpatient and outpatient medical claim diagnosis fields using relevant ICD-9 and ICD-10 codes. A variable to represent the magnitude and burden of comorbidities was calculated using the Charlson Comorbidity Index (CCI) score assessed during the baseline period [15].

Outcomes were assessed using medical and pharmacy claims during the post-index period, and included: (1) total medication days; (2) proportion of days covered (PDC); (3) adherence status; (4) persistence to initiating PD medication therapy; and (5) time to non-persistence of initiating PD medication therapy.

Total medication days for each patient was calculated for the initiating medication claim through the end of the post-index period. We treated all DA claims as a class and grouped claims together, regardless of the drug ingredient that was dispensed within that particular drug class. Total medication days was calculated by summing each indicated day of drug possession for the post-index period.

The PDC was calculated by dividing total medication days by 365 for each patient [16], [17]. Adherence status was assigned to each patient based on an assessment of the calculated PDC. Patients with a PDC of 0.8 or greater were classified as “adherent.”

Persistence was determined by identifying the last consecutive medication day of the initiating medication during the post-index period, without a gap in therapy of 60 days or greater. Given that we allowed for a 60-day runout period to determine a gap in therapy, persistence was assessed through day 305 of the post-index period. Therefore, if the last day of persistence was less than 305, the patient was classified as non-persistent.

### 3.4 Statistical analyses

Potential treatment selection bias was addressed by using stabilized inverse probability of treatment weighting (sIPTW) in our comparative analyses [18]. We first calculated propensity scores with a binary logistic regression model to estimate likelihood of treatment with levodopa or a DA using gender, age, CCI score, and baseline comorbid conditions as independent variables. We created the sIPTW weights by calculating the inverse of the probability of treatment and multiplying by the expected value of the utilization of the treatments in our sample. We assessed cohort balance on the baseline variables using standardized differences between the adjusted study cohorts. We then constructed adjusted (sIPTW-weighted) multivariable linear (dependent variable = PDC) and logistic (dependent variable = adherence status) regression models using the same baseline variables noted above as covariates.

An adjusted Kaplan-Meier survival curve was generated to assess persistence to the initiating PD medication over the post-index period and an adjusted Cox proportional hazards model was constructed to calculate hazard ratios to time to non-persistence between the levodopa and DA cohorts, adjusting for all other covariates.

All statistical analyses were two-tailed, and the significance level was set a priori at p < 0.05, with confidence intervals set at 95%. We conducted supplemental analyses using robust standard errors within our regression models to assess the impact of using an alternative variance estimation method due to our weighting approach. Analyses were performed using IBM SPSS Statistics for Windows, Release 26, Stata Statistical Software, Release 16, and SAS 9.4.

## 4 Results

### 4.1 Patient population

Our study sample of 805 Medicaid patients meeting all inclusion criteria was representative of a younger PD population, with an average age of 54.1 years (Table 1) before sIPTW adjustment. Unadjusted gender distribution across the sample was fairly balanced, with 52.0% being female. Levodopa was the predominant PD medication at initiation, accounting for 75.4% of patients (n = 607), resulting in a 3:1 ratio across the study cohort. Unadjusted descriptive analyses showed that levodopa patients were more likely to be male (50.1% vs. 41.4%, p = 0.034) and older (54.8 years vs. 52.1 years, p < 0.001), with a higher overall comorbidity burden (CCI score of 2.75 vs. 2.23, p = 0.005) when compared to DA patients.

**Table 1:** Unadjusted and Adjusted Baseline Characteristics and Descriptive Results for Medication Adherence and Persistence.

Characteristic	Unadjusted			Adjusted (sIPTW)		
	Levodopa Patients (n = 607)	DA Patients (n = 198)	Standardized Difference	Levodopa Patients	DA Patients	Standardized Difference
*Baseline Characteristics*						
Females, n (%)	303 (49.9%)	116 (58.6%)	0.175	51.7%	50.5%	0.024
Mean Age (sd)	54.8 (8.8)	52.1 (9.5)	0.300	54.1 (9.5)	53.9 (8.3)	0.017
Mean Charlson Comorbidity Index Score (sd)	2.75 (2.6)	2.23 (2.1)	0.207	2.62 (2.6)	2.60 (2.3)	0.008
Baseline Depressive Disorder, n (%)	309 (50.9%)	103 (52.0%)	0.022	51.0%	50.0%	0.020
Baseline Anxiety Disorder, n (%)	227 (37.4%)	90 (45.5%)	0.165	39.2%	38.9%	0.006
Baseline Dementia, n (%)	86 (14.2%)	14 (7.1%)	0.232	12.4%	12.6%	0.006
Baseline Psychosis, n (%)	91 (15.0%)	30 (15.2%)	0.006	15.2%	16.7%	0.041
Baseline Insomnia, n (%)	56 (9.2%)	23 (11.6%)	0.079	9.9%	10.1%	0.007
Baseline Cognitive Impairment, n (%)	ǂ	ǂ	–	ǂ	ǂ	–

			p value			p value
*Medication Adherence*						
Mean Medication Days (sd)	228.2 (117.7)	196.4 (124.8)	0.002	226.8 (117.7)	199.3 (125.2)	0.007
Mean PDC (sd)	0.625 (0.323)	0.538 (0.342)	0.002	0.621 (0.322)	0.546 (0.343)	0.007
Adherent^a^ Patients, n (%)	254 (41.8%)	65 (32.8%)	0.024	41.4%	33.8%	0.060

*Medication Persistence*^b^						
Mean Persistent Days (sd)	210.2 (114.3)	184.5 (118.1)	0.007	209.5 (114.2)	185.4 (117.8)	0.011
Persistent Patients, n (%)	315 (51.9%)	77 (38.9%)	0.002	51.4%	40.6%	0.008

PDC = Proportion of Days Covered.

^ǂ^Cell-size restriction for reporting (<11), balance achieved based on adjusted sIPTW.

sIPTW = stabilized inverse probability weighting.

a Defined as PDC >= 0.80.

b Allowed a gap in therapy of less than 60 days; measured through day 305 of the post-index period.

Unadjusted baseline comorbidities were largely similar across the PD medication cohort groups (Table 1). More than half of all patients (51.2%) had a baseline depressive disorder at time of PD medication initiation. In addition, patients with a baseline anxiety disorder represented 39.4% of the population, with DA patients having a higher prevalence compared to levodopa patients (45.5% vs. 37.4%, p = 0.044). Dementia, psychosis, insomnia, and cognitive impairment comorbidities showed relatively low prevalence across the PD medication cohorts at baseline. Of note, levodopa patients had twice the rate of dementia at initiation of PD therapy compared to DA patients (14.2% vs. 7.1%, p = 0.009).

Based on our assessment of standardized differences in baseline covariates being <0.10 between the adjusted study cohorts as a result of the sIPTW approach, we achieved balance in the treatment cohorts.

### 4.2 Medication adherence

In bivariate analyses, levodopa patients had higher mean medication days translating into a higher mean PDC (Table 1). The adjusted multivariable linear regression model confirmed the significantly higher PDC for levodopa patients versus DA patients (p = 0.005) (Table 2). Female patients had a significantly lower PDC in the post-index period after adjustment of model covariates (p = 0.036). None of the other model covariates indicated a significant relationship with PDC.

**Table 2:** Results from sIPTW-Adjusted Multivariable Linear Regression Model Estimating PDC.

Parameter^a^	Model Coefficient	95% CI	p value
Levodopa Cohort	0.075	0.023–0.128	0.005
Female	−0.050	−0.096 to −0.003	0.036
Age at Index Date	0.002	−0.001 to 0.004	0.153
Charlson Comorbidity Index Score	0.002	−0.008 to 0.012	0.687
Baseline Depressive Disorder	0.025	−0.024 to 0.075	0.311
Baseline Anxiety Disorder	0.010	−0.039 to 0.060	0.687
Baseline Dementia	0.064	−0.010 to 0.137	0.089
Baseline Psychosis	0.000	−0.066 to 0.066	0.993
Baseline Insomnia	−0.043	−0.119 to 0.034	0.277
Baseline Cognitive Impairment	−0.034	−0.124 to 0.057	0.465
Model Constant	0.447	0.303–0.591	< 0.001

Dependent variable = Proportion of Days Covered (PDC).

^a^Reference categories: DA cohort; male; no evidence of baseline comorbidities.

sIPTW = stabilized inverse probability of treatment weighting.

CI = Confidence Interval.

While the unadjusted bivariate analysis of the proportion of adherent patients between levodopa and DA cohorts showed higher rates in the levodopa group, the logistic regression model showed no significant difference in rates between the two groups (p = 0.058) (Table 3).

**Table 3:** Results from sIPTW-Adjusted Logistic Regression Model Estimating Adherence Status.

Parameter^a^	Odds Ratio	95% CI of Odds Ratio	p value
Levodopa Cohort	1.387	0.989–1.947	0.058
Female	0.749	0.559–1.005	0.054
Age at Index Date	1.009	0.993–1.026	0.274
CCI Score	1.016	0.956–1.079	0.610
Baseline Depressive Disorder	1.252	0.918–1.707	0.156
Baseline Anxiety Disorder	1.010	0.738–1.382	0.950
Baseline Dementia	1.395	0.885–2.199	0.152
Baseline Psychosis	1.068	0.706–1.614	0.756
Baseline Insomnia	0.795	0.486–1.301	0.361
Baseline Cognitive Impairment	0.942	0.533–1.663	0.836
Model Constant	0.294	0.115–0.753	0.011

Dependent variable = Adherent patient (defined as PDC >= 0.80).

CCI = Charlson Comorbidity Index; CI = Confidence Interval.

^a^ Reference categories: DA cohort; male; no evidence of baseline comorbidities.

### 4.3 Medication persistence

Bivariate analyses showed that levodopa patients had significantly higher rates of medication persistence compared to DA patients during the first 305 days of the post-index period. Slightly more than half (51.4%, adjusted) of levodopa patients continued use of the medication, without a gap of 60 days or greater, while only 40.6% (adjusted) of DA patients were persistent in medication use (p = 0.008) (Table 1). Levodopa patients averaged 209.5 days of persistence over the 305-day period, compared to 185.4 days for DA patients (p = 0.011).

**Fig. 1:**
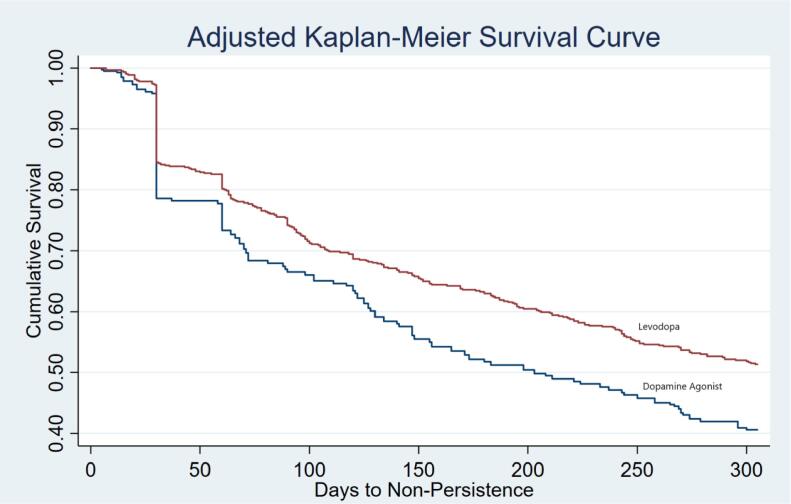
Adjusted Kaplan-Meier Survival Curve to Assess Time to Non-Persistence Dependent variable = Time to Non-Persistence; Allowed a gap in therapy of less than 60 days and measured through day 305 of the post-index period; Log Rank Chi Square Test, p = 0.006.

An adjusted Kaplan-Meier curve showed consistently higher rates of persistence for levodopa patients over the observation period (Fig. 1). A log rank test confirmed significance between the two curves (p = 0.006). Of particular note, the curve shows a large decline in rates of persistence for both levodopa and DA patients at 30 days post-index, with a smaller decline at 60 days.

The adjusted Cox proportional hazards model showed a 26% lower risk of non-persistence for levodopa patients versus DA patients (HR 0.740, CI 0.597–0.917, p = 0.006) (Table 4). Following a similar directional trend seen in the adherence outcome, females had a 23.8% higher risk of non-persistence with their PD medication therapy (HR 1.238, CI 1.015–1.509, p = 0.035). No other factors in the model were found to have a significant relationship with the risk of non-persistence. Finally, we found the use of robust standard errors in the regression models in supplementary analyses did not produce any meaningful difference in our results.

**Table 4:** Results from sIPTW-Adjusted Cox Proportional Hazards Model Estimating Time to Non-Persistence.

Parametera	Hazard Ratio	95% CI of Hazard Ratio	p value
Levodopa Cohort	0.740	0.597–0.917	0.006
Female	1.238	1.015–1.509	0.035
Age at Index Date	0.995	0.985–1.006	0.406
CCI Score	0.987	0.946–1.030	0.544
Baseline Depressive Disorder	0.901	0.730–1.111	0.328
Baseline Anxiety Disorder	0.946	0.766–1.167	0.603
Baseline Dementia	0.752	0.536–1.055	0.099
Baseline Psychosis	1.012	0.761–1.347	0.933
Baseline Insomnia	1.280	0.939–1.744	0.118
Baseline Cognitive Impairment	1.214	0.835–1.764	0.310

^a^Reference categories: DA cohort; male; no evidence of baseline comorbidities.

Allowed a gap in therapy of less than 60 days and measured through day 305 of the post-index period.

CCI = Charlson Comorbidity Index; CI = Confidence Interval.

Total failure incidence = 413/805 (51.3%); 163,866 person-days at risk.

## 5 Discussion

Our analysis of Medicaid patient treatment patterns following PD medication initiation provided a unique opportunity to assess utilization and outcomes in a population of PD patients enrolled in state Medicaid program systems of healthcare delivery.

In general, given the adjusted mean age of patients in our sample of 54 years and exclusion of dual-eligible enrollees, our study sample represented relatively younger patients likely in the earlier stages of PD progression. For comparison, the literature reports an average age of onset of 61.6 years based on patient recall of initial symptoms [19] and an average age of diagnosis of 70.5 years using medical record data [20].

Roughly half of the patients in our sample were female, which is slightly lower than the 58% prevalence of nonelderly females in the Medicaid program across all states [21]. Between our study cohorts, females were less likely to initiate therapy with levodopa (49.9%) versus DA (58.6%). The higher proportion of males initiated on levodopa has not been previously reported in the literature and we are not aware of a clinical rationale for this relationship.

Half of our patients in our unadjusted and adjusted samples had evidence of a depressive disorder at baseline, with nearly 40% showing evidence of an anxiety disorder. Recent research has reported that for PD patients with comorbid depressive or anxiety disorder, the onset of the depressive or anxiety disorder occurred prior to the PD diagnosis in over half of patients [22]. Understanding the relationship between the course of neurodegenerative changes as potential predictors of PD in the Medicaid population may be of benefit, leading to an earlier PD diagnosis. In addition, understanding the etiology of PD could result in earlier intervention with treatment modalities to slow disease progression.

We used PDC to estimate adherence to PD medications in the first 12 months of therapy. Our findings that less than half of patients (adjusted) were considered “adherent” to both levodopa (41.4%) and DA (33.8%) largely reflect the adherence rates from 33% to 54% reported by other researchers using commercial and Medicare claims data [9], [10], [11], [12], [13].

Persistence to these medications was also suboptimal over the post-index period, as just over half of levodopa patients and roughly 40% of DA patients remained persistent with medication, without a gap in therapy of 60 days or greater. The significant difference between the levodopa and DA cohorts with respect to persistence may have clinical implications for identifying optimal initial therapies.

Furthermore, the Kaplan-Meier curve identified two intervals (30 and 60 days post-initiation) where incidence of non-persistence occurred most frequently across the observation period. Given that these are the first or second prescription fills of the drug, this may be an indication that issues related to efficacy or tolerability occurred early in treatment within this subset of patients.

Previous research has reported a similar treatment pattern, with more dramatic drops in persistence at 60 days post-index, resulting in 47.2% of patients identified as non-persistent [11]. Studying this group of patients to understand the drivers of non-persistence may provide insight in ensuring optimal outcomes or to develop intervention programs that promote persistence to therapy over this initial period of treatment.

Poor adherence is associated with worsening of motor symptoms and motor complications as well as side effects and a poor response to the prescribed PD medication [23]. Adherence and persistence to therapy also have implications for health system spending. Greater adherence to PD medications has been shown to significantly lower overall healthcare spending for PD patients in private health plans, with non-adherent patients experiencing higher hospitalization and outpatient costs [13]. We would expect lower rates of adherence, as seen within our sample of patients, to have similar effects more broadly across state Medicaid programs.

The implications of non-adherence and non-persistence within the Medicaid population may also have broader impacts for other programs. Given the natural progression of PD and the aging population, we would expect to see a continuation of the cost implications transferred to the Medicare program as enrollees transition across publicly-funded coverage over time.

### 5.1 Limitations

We used pharmacy claims data to assess outcomes for adherence and persistence in the Medicaid population. Therefore, we assumed that all medications dispensed to patients were started on the day of dispensing and were consumed by the patient as prescribed over the time period we calculated. Patients may have had access to medications through physician samples or acquired medications outside of Medicaid program coverage for which we could not account. To the degree any of these utilization measures represent misclassification bias, it would impact our estimates of adherence and persistence at a population level. Even with these considerations, the use of pharmacy claims has been found to be a reliable method to identify medication exposures over time [24].

Secondly, while our analyses relied on data collected from Medicaid programs across 7 states, our findings may not be generalizable to the entire US Medicaid population. We were limited in the disclosure of the states included in our sample due to restrictions from the data provider.

Finally, we did not have access to information that controlled for medical policies (e.g., preferred drug lists, step-therapy, prior-authorization) that were in place across states during our study period that may have impacted access to treatment and resulting utilization patterns of our study medications.

## 6 Conclusion

Adherence and persistence rates were largely suboptimal during the 12 months following initiation of PD medication for patients in Medicaid programs – a population characterized by relatively younger PD patients. While we did not directly assess the impact of adherence and persistence on resource utilization or costs, previous research has shown that low adherence in this patient population has a negative impact on the use of other services and resulting higher costs of care. Therefore, interventions should be encouraged to address factors directly leading to low adherence and persistence rates in Medicaid PD patient populations. In addition, strategies to address treatment gaps that remain may include the development of alternative PD therapies that provide beneficial efficacy along with limiting side effects to more positively impact adherence and persistence rates.

## Financial disclosures

Drs. Johnsrud and Richards are employees of The University of Texas at Austin and conducted the research through a grant from Cerevel Therapeutics. Drs. Arcona, Sasané, and Leoni are employees of Cerevel Therapeutics.

## Funding

Financial support for the study was provided by Cerevel Therapeutics.

## Author Contributions

All authors contributed to study design, interpretation of data, review of the manuscript, and the decision to submit the article for publication. Drs. Johnsrud and Richards also contributed to the data curation and analyses, and initial draft of the manuscript.

## CRediT authorship contribution statement

**Michael Johnsrud:** Conceptualization, Methodology, Investigation, Data curation, Formal analysis, Writing - original draft, Writing - review & editing. **Kristin Richards:** Conceptualization, Methodology, Investigation, Data curation, Formal analysis, Writing - original draft, Writing - review & editing. **Steve Arcona:** Conceptualization, Methodology, Investigation, Writing - review & editing. **Rahul Sasané:** Conceptualization, Methodology, Investigation, Writing - review & editing. **Matthew Leoni:** Conceptualization, Methodology, Investigation, Writing - review & editing.

## Declaration of Competing Interest

The authors declare that they have no known competing financial interests or personal relationships that could have appeared to influence the work reported in this paper.
